# A Novel Lipid-Based MALDI-TOF Assay for the Rapid Detection of Colistin-Resistant *Enterobacter* Species

**DOI:** 10.1128/spectrum.01445-21

**Published:** 2022-02-02

**Authors:** Richard D. Smith, Christi L. McElheny, Jerilyn R. Izac, Francesca M. Gardner, Courtney E. Chandler, David R. Goodlett, Yohei Doi, J. Kristie Johnson, Robert K. Ernst

**Affiliations:** a Department of Microbial Pathogenesis, University of Maryland Baltimore, Baltimore, Maryland, USA; b Department of Pathology, University of Maryland, Baltimore, Maryland, USA; c Division of Infectious Diseases, University of Pittsburgh School of Medicine, Pittsburgh, Pennsylvania, USA; d Department of Microbiology, Fujita Health University, Toyoake, Aichi, Japan; e Department of Infectious Diseases, Fujita Health University, Toyoake, Aichi, Japan; f Department of Biochemistry & Microbiology, University of Victoria, Victoria, British Columbia, Canada; g University of Gdansk, International Centre for Cancer Vaccine Science, Gdansk, Poland; Weill Cornell Medicine

**Keywords:** *Enterobacter*, antibiotic resistance, diagnostics, mass spectrometry, polymyxins

## Abstract

*Enterobacter* species are classified as high-priority pathogens due to high prevalence of multidrug resistance from persistent antibiotic use. For *Enterobacter* infections caused by multidrug-resistant isolates, colistin (polymyxin E), a last-resort antibiotic, is a potential treatment option. Treatment with colistin has been shown to lead to emergence of polymyxin resistance. The primary mechanism for colistin resistance is modification of terminal phosphate moieties of lipid A, leading to decreased membrane electronegativity and reducing colistin binding affinity. Detection of these modifications, including the addition of phosphoethanolamine and 4-amino-4-deoxy-l-arabinose (Ara4N), can be used for prediction of colistin resistance using matrix-assisted laser desorption ionization-time of flight mass spectrometry (MALDI-TOF MS). The objective of this study was to identify lipid A markers for colistin resistance in *Enterobacter* species and Klebsiella aerogenes (formerly Enterobacter aerogenes). Using a collection of *Enterobacter* and Klebsiella aerogenes clinical isolates, broth MICs for colistin were determined initially. Subsequently, killing assays were carried out to determine how the concentration of colistin at which there is approximately 50% survival (kill_50_) equates to their MICs. Finally, lipid A analysis was conducted via MALDI-TOF MS using the novel rapid extraction method, termed fast lipid analysis technique (FLAT), to correlate MIC and killing efficacy with predictive lipid A modifications. Sensitivity and specificity of the MS assay compared to MIC interpretation were 100% and 53.4%, respectively. A receiver operator characteristic (ROC) demonstrated that MS was highly correlated with killing, with area under the curve of 0.97. This analysis demonstrated the potential utility of MALDI-TOF MS as a rapid diagnostic platform of colistin resistance in *Enterobacter* species.

**IMPORTANCE** In this study, we develop a novel method for identifying colistin resistance in *Enterobacter* species and Klebsiella aerogenes without performing antimicrobial susceptibility testing. Typically, susceptibility testing requires an additional 24 to 48 h, while the MS assay described in this study allows for resistant identifications in under 1 h after initial culture. Identification using MALDI-TOF MS would save time and prevent inappropriate use of colistin. MALDI-TOF MS is an easy-to-use, readily available, robust diagnostic tool in clinical laboratories. Furthermore, this study highlights limitations of polymyxin susceptibility testing. Use of a killing assay best captures how colistin treats infection and is shown to be highly correlated with our MS assay; thus, the MS assay in this study effectively predicts how colistin would treat a patient’s infection. Use of MALDI-TOF MS for accurate and early identification of antimicrobial resistance can improve antimicrobial stewardship and patient outcomes.

## INTRODUCTION

*Enterobacter* is a genus of Gram-negative rod-shaped bacteria and part of the *Enterobacteriaceae* family (1). A common health care-associated pathogen, *Enterobacter* species can cause infections in the respiratory tract, urinary tract, and surgical wounds (2). Additionally, *Enterobacter* species are a common cause of hospital-acquired bacteremia. *Enterobacter* species are included in the list of ESKAPE (Enterococcus faecium, Staphylococcus aureus, Klebsiella pneumoniae, Acinetobacter baumannii, Pseudomonas aeruginosa, and *Enterobacter* species) pathogens, common multidrug-resistant organisms that are considered highest priority for developing new antimicrobial therapies due to increased levels of resistance, virulence, and lack of available therapies by both the CDC and WHO (3, 4). Colistin is one of the few active antibiotics for treatment of multidrug-resistant (MDR) *Enterobacter* infections (5). However, the emergence and increasing incidence of colistin resistance have led to further clinical complications in treating infections due to a reduced number of viable treatment options (5).

Colistin, or polymyxin E, is a cationic lipopeptide and “last-resort” antibiotic used for MDR Gram-negative infections, such as those producing extended-spectrum beta-lactamases and those resistant to carbapenems (6), which is an increasing concern in clinical isolates of *Enterobacter* species (7). Targeting the cell membrane, cationic colistin binds with anionic lipopolysaccharide (LPS) through electrostatic interactions (8–11). Colistin resistance is conferred by reduction of the electronegativity of the cell membrane through modification of the terminal phosphate residues of lipid A, the membrane anchor of LPS, thereby decreasing the binding affinity of colistin (12). Lipid A modifications that reduce electronegativity of the cell membrane include the addition of phosphoethanolamine and 4-amino-4-deoxy-l-arabinose (Ara4N), which have been detailed in longitudinal studies for colistin-resistant Escherichia coli, Acinetobacter baumannii, Pseudomonas aeruginosa, and Klebsiella pneumoniae (13–17). With increasing usage of colistin, increasing levels of resistance of P. aeruginosa have been observed, especially in patients with cystic fibrosis (18). The growing prevalence of colistin-resistant pathogens has created a significant demand for the development of a rapid diagnostic to differentiate resistant isolates from intermediate or sensitive isolates.

Currently, phenotypic antibiotic susceptibility testing (AST) is the standard method for identification of resistance in pathogens and is performed via broth microdilution or disc diffusion assay to determine the MIC. The MIC is the lowest concentration of antibiotic where growth inhibition is observed. Testing for colistin susceptibility using these classic methods is problematic (19, 20). Lack of reproducibility and inconsistencies in results depending on testing methods and bacterial growth parameters encompass some of the limitations of the methodology (21–23). Broth dilution is favored, as colistin diffuses poorly in agar, but is cumbersome to perform in a clinical laboratory setting (24, 25). According to the Clinical and Laboratory Standards Institute (CLSI), colistin breakpoint for *Enterobacterales* intermediate susceptibility is defined as an MIC of 2 μg/mL or less, and greater than 2 μg/mL is defined as resistant (26). For the European Committee on Antimicrobial Susceptibility Testing (EUCAST), an MIC of 2 μg/mL or less is defined as susceptible and greater than 2 μg/mL is defined as resistant (27).

Matrix-assisted laser desorption ionization-time of flight mass spectrometry (MALDI-TOF MS) is one of the prominent technologies in clinical microbiology laboratories. MALDI-TOF MS provides simple, rapid, and robust sample preparation (28). Two MALDI-TOF MS platforms commonly used in clinical laboratories are the Bruker MALDI Biotyper (MBT) (Bruker Daltonics, Billerica, MA) and bioMérieux Vitek MS (bioMérieux, Inc., Durham, NC) (28). Both protein-based methods utilize an extensive library of mass spectra mainly from ribosomal proteins, highly conserved in bacterial species, as a reference to identify organisms (29). Major drawbacks of these protein-based MALDI-TOF MS platforms are their limited capabilities detecting polymicrobial infections and antimicrobial resistance (30). However, analyzing glycolipids (lipid-based) by MS adds the ability to detect polymicrobial infections and antimicrobial resistance, especially those affecting the bacterial cell envelope, including colistin, in an easy, rapid, and reproducible manner (13–17, 30).

Previous studies have demonstrated the use of microbial membrane glycolipids for the rapid identification of pathogens, including Gram-negative and Gram-positive bacteria and fungi (13–17, 31, 32). Recently, our group has developed a novel lipid extraction method, fast lipid analysis technique (FLAT), which provides potential utility for clinical microbiology laboratory settings (31). FLAT involves the extraction of lipids directly on a MALDI plate in less than 1 h, significantly reducing labor and handling time compared to those of previous lipid analysis methods. Additionally, FLAT allows for identification of antimicrobial resistance via a MALDI-TOF MS platform. Furthermore, we have shown that modifications to lipid A in the ESKAPE pathogens A. baumannii, K. pneumoniae, and P. aeruginosa can be used to determine colistin resistance detection via MALDI- TOF MS analysis. For example, addition of phosphoethanolamine, l-amino-4-arabinose (Ara4N), or galactosamine to terminal phosphates of lipid A reduces membrane electronegativity reducing permeability, thus conferring colistin resistance (13–17). Presence of all these modifications has been observed via MALDI-TOF MS. Finally, mobilized colistin resistance (MCR) genes confer plasmid-mediated resistance to colistin. The mechanism of this resistance is a phosphatidylethanolamine transferase which transfers phosphoethanolamine to lipid A (33). For all Gram-negative ESKAPE pathogens, *mcr* genes and other mechanisms of colistin resistance have been detected and through horizontal gene transfer have rapidly spread to other Gram-negative pathogens. Therefore, a rapid, direct-from-samples analysis is critical for global surveillance of colistin-resistant organisms (16, 34, 35).

Previous studies have confirmed the ability to analyze glycolipids via MS to identify colistin resistance in the other Gram-negative ESKAPE pathogens. This study sought to analyze this using *Enterobacter* species and Klebsiella aerogenes after a recent surveillance study by the British Society for Antimicrobial Chemotherapy identified that the annual colistin resistance rates among E. cloacae complex isolates were 4.4% to 20%, which was considerably higher that the <2% for other Gram-negative *Enterobacteriaceae* organisms (36). Furthermore, this study was the first to confirm the use of FLAT as a rapid, clinically viable assay for the determination of colistin resistance compared to standard broth microdilution AST methods.

## RESULTS

### *Enterobacter* species clinical isolate overview.

Ninety-eight *Enterobacter* species isolates were collected from 2011 to 2017, of which 74 were Enterobacter cloacae complex organisms and 24 were identified as *Klebsiella* (*Enterobacter*) *aerogenes*. For the Enterobacter cloacae complex organisms, the following species were identified: E. cloacae, E. asburiae, E. hormaechei, E. kobei, E. ludwigii, and E. nimipressuralis (1). K. aerogenes isolates were included in this study since they were initially classified as Enterobacter aerogenes and treated as *Enterobacter* infections during the study period. Furthermore, K. aerogenes has numerous phenotypic traits shared with *Enterobacter* (37, 38). A schematic breakdown of all clinical isolates, MIC interpretations, and MS results is displayed in Fig. S1.

### Determination of MIC levels for *Enterobacter* clinical isolates.

MICs ranged from 0 μg/mL to 128 μg/mL with replicate analysis consistent for all isolates. Of the 98 total isolates, 88 were classified intermediate (CLSI) or susceptible (EUCAST) (≤2 μg/mL) and 10 resistant to colistin (>2 μg/mL). A breakdown for all isolates was as follows: 10 isolates displayed an MIC of 0.125 μg/mL, 14 of 0.25 μg/mL, 302 of 0.5 μg/mL, 23 of 1 μg/mL, 9 of 2 μg/mL, 1 of 4 μg/mL, 1 of 32 μg/mL, 2 of 64 μg/mL, and 6 of 128 μg/mL.

### Observed lipid A modifications in Enterobacter species and Klebsiella aerogenes by MALDI-TOF MS.

**FIG 1:**
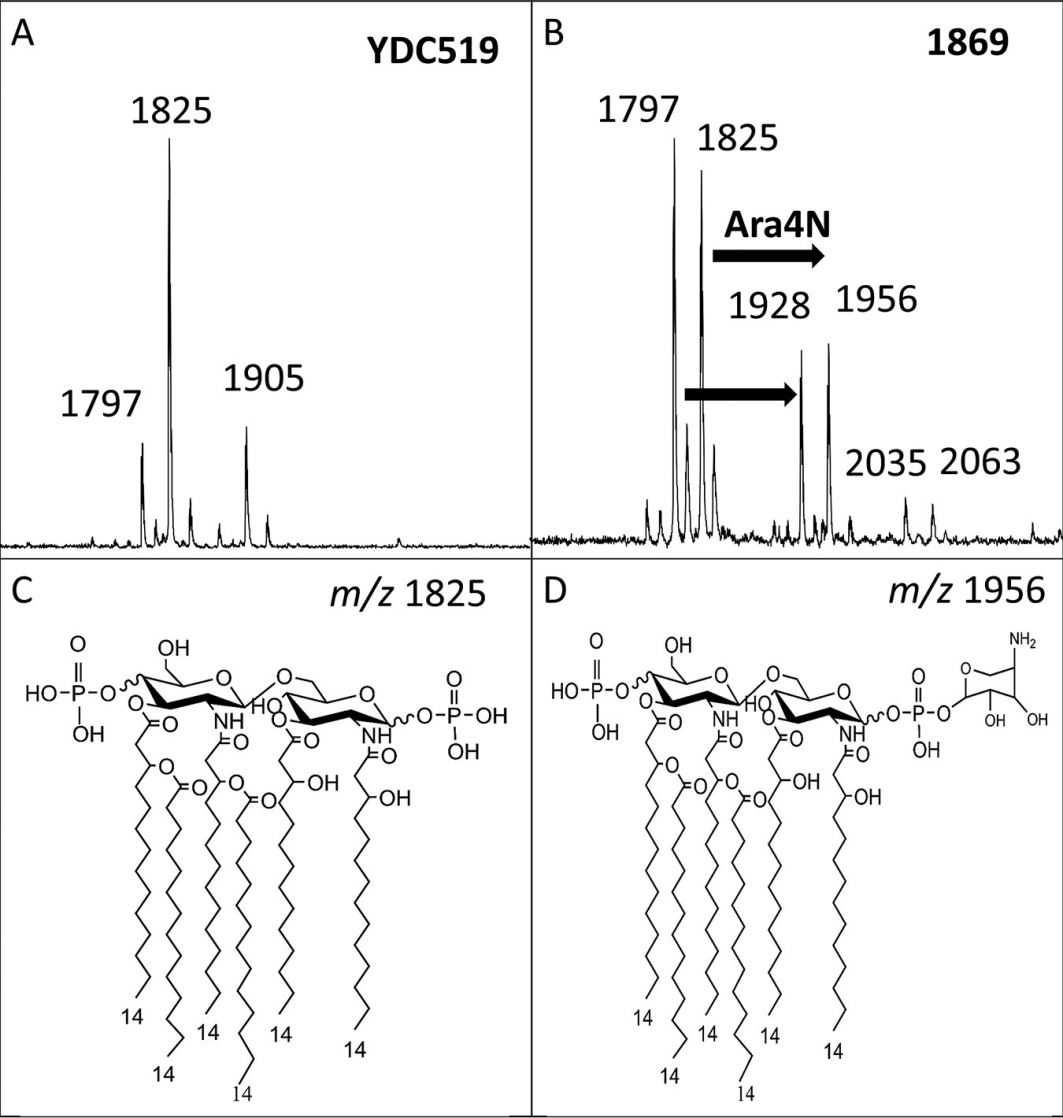
MALDI-TOF MS of *Enterobacter* species and Klebsiella aerogenes with difference in colistin susceptibility. Isolates from patients YDC519 and 1869 were grown for 16 h at 37°C on LB agar plates with 2 μg/mL of colistin and without colistin. (A) YDC519 is a colistin-susceptible isolate and has a base lipid A structure consistent with *m/z* 1,825 along with a two-carbon reduction of an acyl chain (*m/z* 1,797) and phosphorylation (*m/z* 1,905). (B) Isolate 1869 is colistin-resistant with *m/z* 1,825 and *m/z* 1,797 as base peaks. Additional ions at *m/z* 1,928 and *m/z* 1,956 indicate an Ara4N addition to base structures. Palmitoylation is shown (*m/z* 2,035, *m/z* 2,063). (C) Lipid A structure for base peak for *Enterobacter.* (D) Lipid A structure commonly found in colistin-resistant isolates.

**FIG 2:**
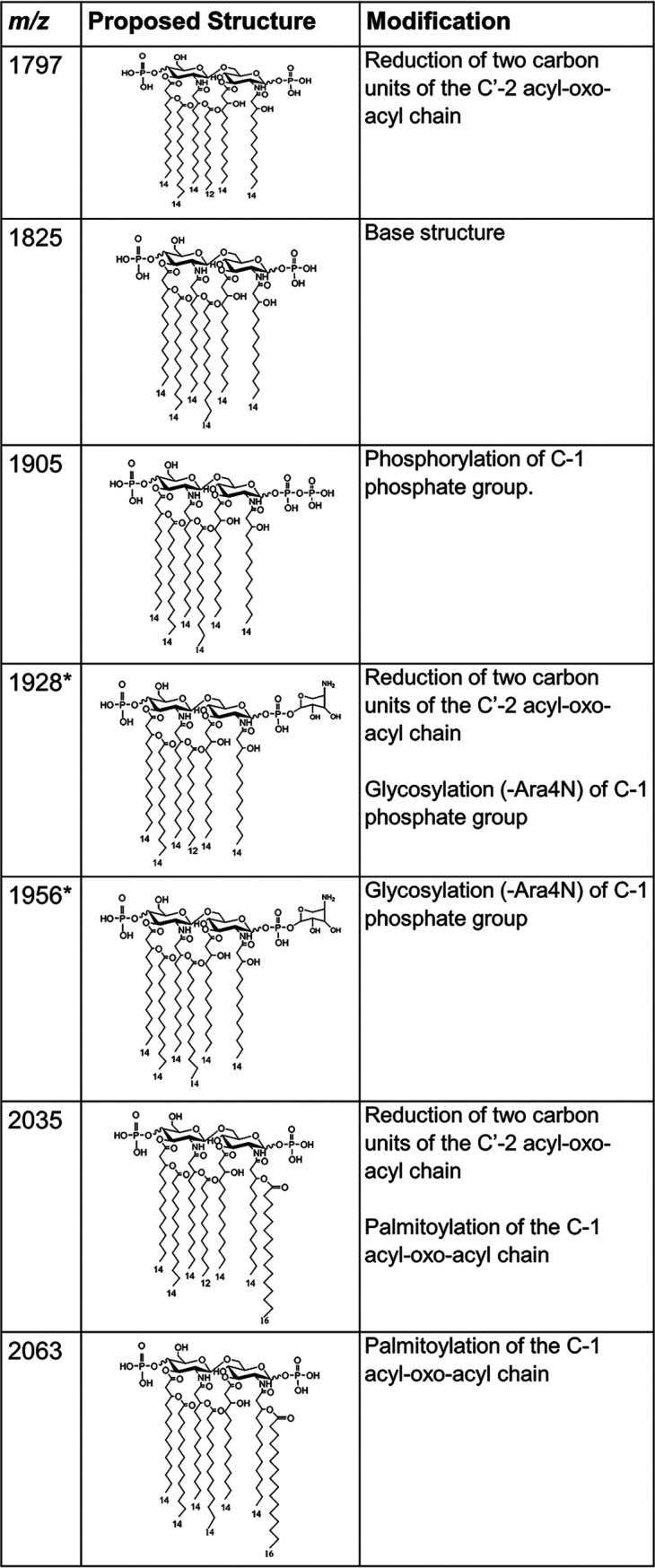
Lipid A structures with respective *m/z* values commonly found in clinical isolates of *Enterobacter* and Klebsiella aerogenes. Modifications causing observed mass shifts are described. Asterisks indicate ion associated with colistin resistance.

As shown in Fig. 1, we identified the base lipid A ion (*m/z* 1,825), which has previously been reported and is indicative of *Enterobacter* species (32). This ion represents a bisphosphorylated hexa-acylated structure (Fig. 2). Additional ions at *m/z* 1,905 and *m/z* 2,063 represent the addition of a third phosphate (Δ*m/z* 80) or palmitate (C_16_, Δ*m/z* 238), respectively (Fig. 2). The minor ions of *m/z* 1,797 and 2,035 represent a two-carbon reduction at the C′-2 acyl-oxo-acyl chain for the respective ions of *m/z* 1,825 and 2,063 (C_2_H_4_, Δ*m/z* 28). The minor ion at *m/z* 1,928 represents a two-carbon reduction at the C′-2 acyl-oxo-acyl chain for the respective ions of *m/z* 1,956 (C_2_H_4_, Δ*m/z* 28). Finally, the ion at *m/z* 1,956 represents the base structure with the addition of an Ara4N moiety (Δ*m/z* 131) to one of the terminal phosphate groups (Fig. 1) and is a signature biomarker for colistin resistance. Of the 98 isolates, 51 isolates had the ion at *m/z* 1,956 present.

### Correlation of MIC and lipid A modification of *Enterobacter* species.

When screening *Enterobacter* species and Klebsiella aerogenes isolates by MALDI-TOF MS to identify potential mechanisms of colistin resistance, we observed addition of Ara4N (Δ*m/z* 131), a mechanism of colistin resistance that has been reported previously (39). All isolates were screened by MS for presence of Ara4N and compared to current CLSI and EUCAST MIC standard thresholds. For all 10 isolates with a resistant MIC, Ara4N addition was observed by MS analysis. This yielded a sensitivity of 100%, demonstrating that Ara4N is a highly sensitive biomarker for identifying colistin resistance. Of the 88 isolates determined with an intermediate MIC, 41 of the isolates were determined resistant by MS with Ara4N present, and in 47 isolates the Ara4N was absent and determined intermediate by MS. Based on these results, the specificity for the MS-based colistin assay was 53.4%. Mean and median MICs for the Ara4N present and absent groups were compared. Median MICs for Ara4N present and absent groups were 1.20 (interquartile range [IQR]: 0.50, 1.50) and 0.50 (IQR: 0.12, 1.00), respectively. The mean MICs for Ara4N present and absent were 18.8 μg/mL (95% confidence interval [CI]: 7.40, 30.2) and 0.69 (95% CI: 0.52, 0.86), respectively (Table 1).

**TABLE 1:** Concentrations of colistin to kill 50% of the bacterial population (kill_50_) for all isolates, resistant isolates, and intermediate resistant isolates, comparing isolates with Ara4N and without with 95% confidence interval (CI); furthermore, comparison of median and mean MIC between Ara4N present and absent groups^*a*^

Parameter	Colistin concn μg/mL (95% CI)		*P* value
	Ara4N present	Ara4N absent	
Isolate			
All isolates	6.37 (5.97, 7.71)	0.19 (0.12, 0.28)	<0.001
Resistant (>2 μg/mL)	9.12 (8.21, 12.5)	NA	NA
Intermediate (≤2 μg/mL)	3.55 (2.40, 4.38)	0.19 (0.12, 0.28)	<0.001
MIC			
Median MIC	1.20 (0.50, 1.50)	0.50 (0.12, 1.00)	0.021
Mean MIC	18.8 (7.40, 30.2)	0.69 (0.52, 0.86)	<0.001

a For each group, significant differences were seen comparing those with Ara4N to those without (*P* < 0.001).

### Killing assay.

**FIG 3:**
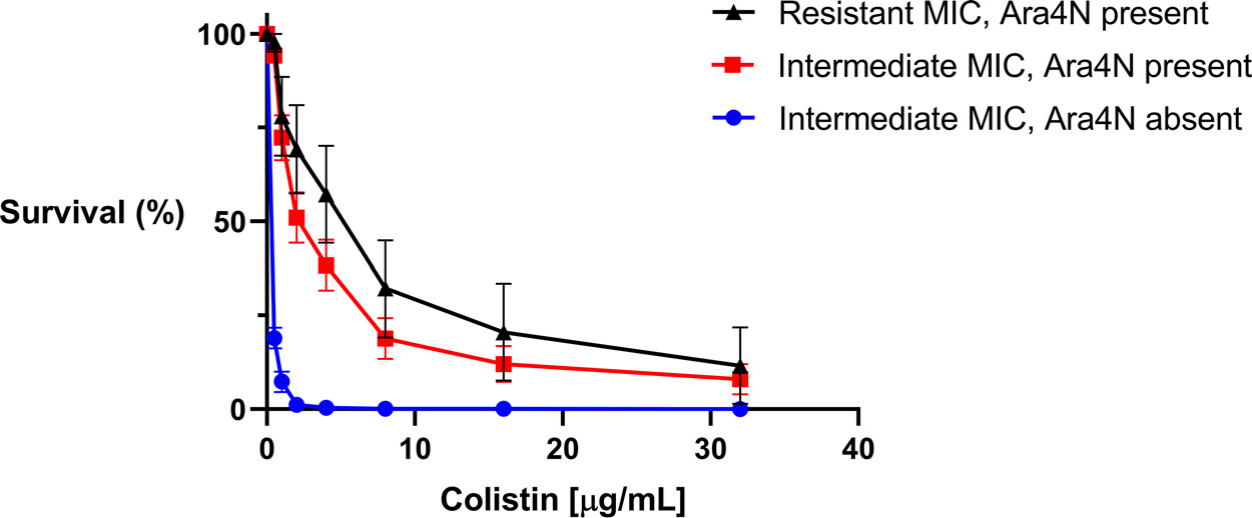
Killing assay comparing intermediate resistant isolates by MIC/Ara4N present (*n* = 41), intermediate isolates by MIC/Ara4N absent (*n* = 47), and resistant isolates by MIC/Ara4N (*n* = 10) present percent survival after 30 min of killing at different colistin concentrations. These curves were statistically different by two-way ANOVA (*P* < 0.001).

Percent survival at different colistin concentrations was plotted to compare isolates with and without Ara4N (Fig. 3). The average concentration of colistin at which there was approximately 50% survival (kill_50_) for all isolates with Ara4N present was 6.37 μg/mL (95% CI: 5.97, 7.71) and 0.19 μg/mL (95% CI: 0.12, 0.28) for those without Ara4N (Table 1). When stratified, intermediate isolates with the modification had an average kill_50_ of 3.55 μg/mL (95% CI: 2.40, 4.38) and 0.19 μg/mL (95% CI: 0.12, 0.28) for those without the Ara4N modification (*P* < 0.001). Kill_50_ for the 10 resistant isolates was 9.12 μg/mL (95% CI: 8.21, 12.49). Plots comparing percent survival at different colistin concentrations for isolates with resistant MICs and Ara4N present, intermediate MICs and Ara4N present, and intermediate MICs and Ara4N absent were significantly different (*P* < 0.001), indicating that the bacterial initial killing kinetics of colistin differ depending on whether Ara4N is present or absent (Fig. 3).

### Receiver operator characteristic curves.

**FIG 4:**
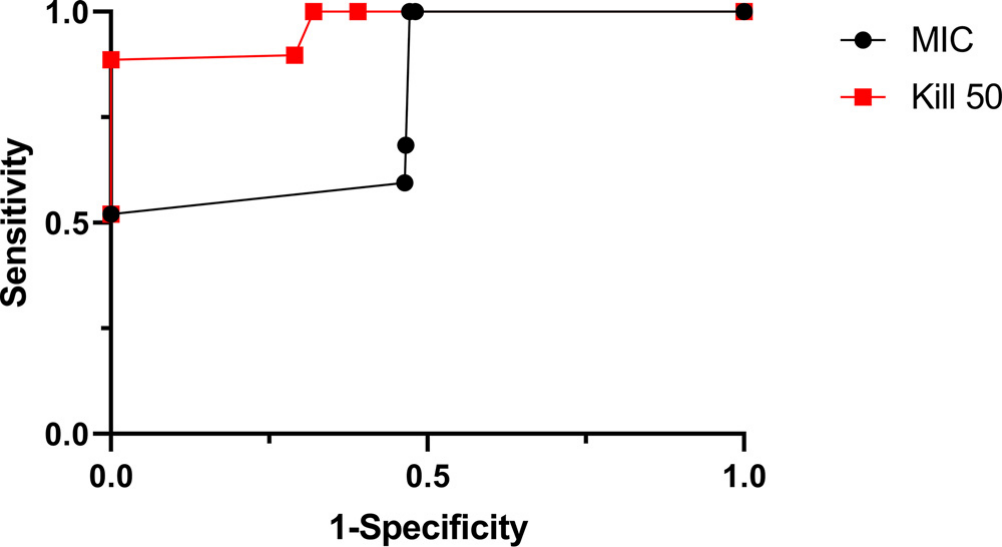
Receiver operator characteristic curves (ROCs) analyzing the diagnostic accuracy of Ara4N compared to MIC (black) and kill_50_ (red). Area under the curve (AUC) for MIC and kill_50_ was 0.74 and 0.97, respectively.

Receiver operator characteristic curves (ROCs) are commonly used to determine optimal thresholds for diagnostic tests and to measure the overall predictive and diagnostic accuracy of a diagnostic test. In this instance, ROCs were produced to assess and compare the overall predictive and diagnostic ability of the MS-based colistin assay compared to that of kill_50_ and MIC (Fig. 4). The area under the curve (AUC) for kill_50_ and broth microdilution was 0.97 and 0.74, respectively. These results indicate that MS identifies initial killing by colistin more accurately than MIC. Furthermore, an AUC of 0.97 illustrates that the MS-based colistin assay with FLAT extraction is strongly associated with kill_50_ and has diagnostic and predictive value when predicting initial killing of colistin. A perfect predictor or diagnostic has an AUC of 1; with an AUC of 0.97, the MS-based colistin assay is an efficient and accurate alternative to capture initial killing of colistin.

### Analysis for MS reproducibility.

The FLAT and MS-base colistin assay were determined to be highly reproducible with zero intraday and interday variability observed among the isolates used. For the five isolates with the Ara4N modification, the modification was observed in all three replicates over the course of 5 days. Furthermore, the modifications remained absent for the four isolates previously determined to have lack Aar4N modification. The absence of the modification was observed for all replicates over the course of 5 days, illustrating the high reproducibility of this assay.

## DISCUSSION

*Enterobacter* species and Klebsiella aerogenes prominently cause health care-associated infections leading to morbidities, such as sepsis and urinary tract infections (UTI) (2). The increasing incidence of antimicrobial resistance has made combatting Enterobacter a top priority by the WHO. To ensure appropriate therapy, it is paramount to develop optimal diagnostic methods. Identification of colistin resistance would further enhance public health outcomes. In this study, using retrospectively collected patient isolates, we observed colistin resistance using MALDI-TOF MS glycolipid analysis, MIC testing, and a colistin killing assay. A strong association between colistin tolerance and an Ara4N addition was observed.

FLAT MS is a highly sensitive method for identification of colistin-resistant *Enterobacter* species and K. aerogenes, identifying resistance for 100% of the isolates with resistance MIC interpretations. However, the limitation of this study is the low specificity, 53.4%. The low specificity of this assay can be explained by the several known challenges with polymyxin AST, especially with *Enterobacter* species and K. aerogenes (40–44). Broth microdilution is currently the “gold standard” for MIC determination since disc diffusion and Etest have higher false-susceptibility rates due to improper diffusion (43). Despite being the gold standard, broth microdilution is also problematic. One major challenge of broth microdilution is colistin adhering to labware causing inaccurate concentrations of colistin in testing wells. In a study with Enterobacter cloacae and Klebsiella aerogenes, Landman et al. (44) demonstrate that polymyxin MICs often have low reproducibility and uninterpretable results. This study observed several inaccurate MIC interpretations, including false susceptibilities along with a “skipped well” phenomenon. This phenomenon is characterized by a well without growth despite growth present at higher concentrations of colistin. A potential explanation for false susceptibilities is the emergence of heteroresistance.

Heteroresistance is defined as having a subpopulation of antibiotic resistance bacteria when the overall population is determined susceptible by *in vitro* methods (45). A public health concern, heteroresistance causes antibiotic treatment failure and negative patient outcomes; thus, it is important to identify (46, 47). Heteroresistance poses a challenge for polymyxin AST, as it leads to misinterpretations and false susceptibilities. Landman et al. demonstrated presence of heteroresistant *Enterobacter* species and a correlation between MIC and proportion of resistant bacteria. Previously, FLAT MS was shown to accurately identify colistin resistance in known heteroresistant populations of E. coli, *Klebsiella* species, and *Enterobacter* species (48). False susceptibilities and the ability of FLAT to identify heteroresistance explain the low specificity of this assay compared to that of traditional colistin AST.

Due to the shortcomings of traditional polymyxin AST and the observed discordance in this study, a killing assay was performed to evaluate the pharmacodynamics of colistin when treating potentially resistant bacteria. Polymyxins kill rapidly through interaction and disruption of the bacterial membrane (49). Because of this, the killing assay best reflects pharmacodynamics and response to treatment with colistin, valuable insight for a clinical setting. Additionally, since colistin is nephrotoxic, short-term treatment is ideal (50). Furthermore, the killing assay does not allow adaptation to better capture heteroresistance and provides enumeration so it is not subject to interpretation like MICs (51). Despite providing important clinical information, killing assays are clinically limited due to high labor requirements and lengthy turnaround times; however, the FLAT MS assay is a near-perfect predictor of killing (AUC = 0.97), making it an appropriate clinical substitute. Since Kill_50_ is significantly higher in isolates where the Ara4N is present, this suggests that treatment efficacy would be worse when the marker is detected.

Ideally, a clinical diagnostic platform should be easy to use, rapid, robust, reproducible, and accurate. For these reasons, MALDI-TOF MS is an important resource in clinical microbiology settings (28). The novel extraction method FLAT paired with MS analysis is easy to conduct, robust, highly reproducible, and accurate, and it provides results in under an hour (31) using instrumentation already present in clinical laboratories. Furthermore, FLAT allows for identification of antimicrobial resistance using a MALDI-TOF MS platform. Further development of this lipid-based diagnostic assay for direct-from-sample analysis, using samples such as urine and blood culture bottles, without the need for *ex vivo* culture further highlights the potential clinical utility of our MALDI-TOF MS diagnostic method.

## MATERIALS AND METHODS

### Bacterial strains.

Collection of clinical isolates (*n* = 98) was approved by the University of Pittsburgh Institutional Review Board (PRO12060302). Isolates included in this study were identified by the clinical microbiology laboratory at the University of Pittsburgh Medical Center via MicroScan WalkAway (Beckman Coulter, Brea, CA) and confirmed by MALDI Biotyper research-use-only (RUO) (Bruker Daltonics, Billerica, MA) as *Enterobacter* species upon arrival to the research laboratory (Table S1). Collected isolates were reported as resistant to carbapenems being tested against ertapenem, meropenem, and imipenem. Isolate collection was conducted for 6 years from 2011 to 2017, and these low passage isolates were glycerol stocked and stored at −80°C until MALDI-TOF MS analysis.

### Susceptibility testing.

MICs were determined by broth dilution method using cationic adjusted Mueller-Hinton broth and conducted following methods published by the CLSI (19). MICs ranged from 0.125 μg/mL to 128 μg/mL. Broth dilutions were replicated 10 times per isolate using separate isolated colonies for each replicate. If there were discordant MICs, broth dilutions were repeated in triplicate. The final MIC was the MIC of the concordant repeated triplicates. Colistin sulfate salt was acquired from Sigma-Aldrich (catalog number C4461; St. Louis, MO). Determination of intermediate and resistant organisms was done using the previously mentioned CLSI breakpoints.

### FLAT lipid A extraction and MALDI-TOF MS analysis.

Isolates were cultured onto lysogeny broth (LB) plates and grown for 16 h at 37°C. To express resistance, samples were also cultured on LB with 2 μg/mL colistin sulfate plates. Isolates with MICs less than 2 μg/mL had reduced growth. Lipid A analysis was conducted using FLAT per the methods in Sorensen et al. (31). Briefly, a single colony was spotted on a MALDI plate, overlaid with 1 μL of buffer of 0.2 M anhydrous citric acid and 0.1 M trisodium citrate dihydrate, incubated at 110°C for 30 min, and rinsed with endotoxin-free water (31). Each isolate was spotted in triplicate. Subsequently, 1 μL of 10 mg/mL norharmane matrix suspended in 2:1 chloroform-methanol was spotted onto the extracted lipid sample on a MALDI plate. A Bruker Microflex LRF MALDI-TOF MS in the negative ion and reflectron mode was used to collect mass spectra. The MALDI-TOF MS comprises a 337 nm nitrogen laser. Analyses were conducted at 43% global intensity with approximately 300 laser shots for each spectrum acquisition. Spectra were recorded in triplicate. Electrospray tuning mix (Agilent, Palo Alto, CA) was used for mass calibration. Flex analysis (v3.4) software processed the mass spectra with smoothed and baseline corrections. Signal-to-noise ratios above three were considered optimal for inclusion of signature ions (52).

### Killing assay.

To determine the kill_50_ for all organisms, individual isolates were grown in 5 mL of lysogenic broth (LB) at 37°C shaking at 180 rpm for 16 h. A subculture was created by adding 250 μL of bacterial culture into 4,750 μL of LB media. Cultures were then grown to 1.5 × 10^8^ CFU/mL (0.5 McFarland standard value) and then diluted to approximately 3 × 10^5^ and 8 × 10^5^ CFU/mL before use. Briefly, 100 μL of this working stock culture was added to 100 μL of LB with colistin at different concentrations and plated in a 96-well round-bottom plate (CELLTREAT Scientific, Pepperell, MA). Concentrations of colistin were 32 μg/mL, 16 μg/mL, 8 μg/mL, 4 μg/mL, 2 μg/mL, 1 μg/mL, 0.5 μg/mL, and 0 μg/mL. Each isolate was plated in triplicate and incubated for 30 min at 37°C to best capture killing at different colistin concentrations, as the bactericidal effect of colistin is extremely rapid. Subsequently, isolates were enumerated for survival by plating on LB and counting cells after 16-h incubation at 37°C. As the negative control, the 0 time point was plated on LB, grown for 16 h at 37°C, and enumerated to measure CFU/mL prior to exposure. Kill_50_ was calculated as follows: cell counts for each isolate at each concentration of colistin were divided by the counts from the 0 time point to calculate percent survival. Percent survival was plotted across each colistin concentration, and the concentration at which there was approximately 50% survival was calculated using nonlinear regression as the kill_50_.

### Analysis for MS reproducibility.

To evaluate reproducibility of the FLAT procedure for colistin resistance identification in *Enterobacter* species and Klebsiella aerogenes, nine isolates, three each with an MIC of 0.5 μg/mL, 2 μg/mL, and 128 μg/mL, were analyzed repeatedly. Of the nine, five showed the presence of Ara4N addition and four lacked this modification during initial analysis. Prior to MS analysis, individual isolates were grown on LB and LB with 2 μg/mL colistin plates (supplemented with 1.5% Bacto-agar). FLAT was conducted for each isolate in triplicate (intraday variability, 27 individual samples) for 5 days (interday variability, 135 individual samples) to evaluate assay reproducibility.

### Statistical analyses.

Sensitivity and specificity comparing MS (presence or absence of Ara4N) and MIC interpretations (intermediate or resistant) were calculated. Comparison of kill_50_ was conducted for all isolates, intermediate isolates only, and resistant isolates only with and without Ara4N. Percent survival was plotted for each group and compared using a two-way analysis of variance (ANOVA). Kill_50_ was calculated for each above group by pooling data from all isolates in each group and fitting a nonlinear regression. Receiver operator characteristic (ROC) curves were created to compare kill_50_, MIC, and specificity and sensitivity of the MS diagnostic at different colistin concentrations. ROC curves were created using thresholds from each tested concentration of colistin for killing assay and broth microdilution. Isolates with Ara4N present were considered positive for resistance and those with Ara4N absent were considered negative. Statistical analyses and graphs were performed using GraphPad Prism (Version 8). Lipid structures were created using Chem Draw (Version 20).

## References

[1] Mezzatesta ML, Gona F, Stefani S. 2012. *Enterobacter cloacae* complex: clinical impact and emerging antibiotic resistance. Future Microbiol 7:887–902. doi:10.2217/fmb.12.61. 22827309

[2] Ronald A. 2003. The etiology of urinary tract infection: traditional and emerging pathogens. Dis Mon 49:71–82. doi:10.1067/mda.2003.8. 12601338

[3] Tacconelli E, Carrara E, Savoldi A, Harbarth S, Mendelson M, Monnet DL, Pulcini C, Kahlmeter G, Kluytmans J, Carmeli Y, Ouellette M, Outterson K, Patel J, Cavaleri M, Cox EM, Houchens CR, Grayson ML, Hansen P, Singh N, Theuretzbacher U, Magrini N, WHO Pathogens Priority List Working Group. 2018. Discovery, research, and development of new antibiotics: the WHO priority list of antibiotic-resistant bacteria and tuberculosis. Lancet Infect Dis 18:318–327. doi:10.1016/S1473-3099(17)30753-3. 29276051

[4] Rice LB. 2008. Federal funding for the study of antimicrobial resistance in nosocomial pathogens: no ESKAPE. J Infect Dis 197:1079–1081. doi:10.1086/533452. 18419525

[5] Osei Sekyere J, Govinden U, Bester LA, Essack SY. 2016. Colistin and tigecycline resistance in carbapenemase-producing Gram-negative bacteria: emerging resistance mechanisms and detection methods. J Appl Microbiol 121:601–617. doi:10.1111/jam.13169. 27153928

[6] Mulani MS, Kamble EE, Kumkar SN, Tawre MS, Pardesi KR. 2019. Emerging strategies to combat ESKAPE pathogens in the era of antimicrobial resistance: a review. Front Microbiol 10:539. 30988669 10.3389/fmicb.2019.00539 PMC6452778

[7] Nikolaidis I, Favini-Stabile S, Dessen A. 2014. Resistance to antibiotics targeted to the bacterial cell wall. Protein Sci 23:243–259. doi:10.1002/pro.2414. 24375653 PMC3945833

[8] Gupta S, Govil D, Kakar PN, Prakash O, Arora D, Das S, Govil P, Malhotra A. 2009. Colistin and polymyxin B: a re-emergence. Indian J Crit Care Med 13:49–53. doi:10.4103/0972-5229.56048. 19881183 PMC2772240

[9] Cetuk H, Anishkin A, Scott AJ, Rempe SB, Ernst RK, Sukharev S. 2021. Partitioning of seven different classes of antibiotics into LPS monolayers supports three different permeation mechanisms through the outer bacterial membrane. Langmuir 37:1372–1385. doi:10.1021/acs.langmuir.0c02652. 33449700

[10] Cetuk H, Maramba J, Britt M, Scott AJ, Ernst RK, Mihailescu M, Cotten ML, Sukharev S. 2020. Differential interactions of piscidins with phospholipids and lipopolysaccharides at membrane interfaces. Langmuir 36:5065–5077. doi:10.1021/acs.langmuir.0c00017. 32306736

[11] Heinrich F, Salyapongse A, Kumagai A, Dupuy FG, Shukla K, Penk A, Huster D, Ernst RK, Pavlova A, Gumbart JC, Deslouches B, Di YP, Tristram-Nagle S. 2020. Synergistic biophysical techniques reveal structural mechanisms of engineered cationic antimicrobial peptides in lipid model membranes. Chemistry 26:6247–6256. doi:10.1002/chem.202000212. 32166806 PMC8146162

[12] Aghapour Z, Gholizadeh P, Ganbarov K, Bialvaei AZ, Mahmood SS, Tanomand A, Yousefi M, Asgharzadeh M, Yousefi B, Kafil HS. 2019. Molecular mechanisms related to colistin resistance in *Enterobacteriaceae*. Infect Drug Resist 12:965–975. doi:10.2147/IDR.S199844. 31190901 PMC6519339

[13] Leung LM, McElheny CL, Gardner FM, Chandler CE, Bowler SL, Mettus RT, Spychala CN, Fowler EL, Opene BNA, Myers RA, Goodlett DR, Doi Y, Ernst RK. 2019. A prospective study of *Acinetobacter baumannii* complex isolates and colistin susceptibility monitoring by mass spectrometry of microbial membrane glycolipids. J Clin Microbiol 57. doi:10.1128/JCM.01100-18. PMC6425172 30567747

[14] Miller AK, Brannon MK, Stevens L, Johansen HK, Selgrade SE, Miller SI, Hoiby N, Moskowitz SM. 2011. PhoQ mutations promote lipid A modification and polymyxin resistance of *Pseudomonas aeruginosa* found in colistin-treated cystic fibrosis patients. Antimicrob Agents Chemother 55:5761–5769. doi:10.1128/AAC.05391-11. 21968359 PMC3232818

[15] Leung LM, Cooper VS, Rasko DA, Guo Q, Pacey MP, McElheny CL, Mettus RT, Yoon SH, Goodlett DR, Ernst RK, Doi Y. 2017. Structural modification of LPS in colistin-resistant, KPC-producing *Klebsiella pneumoniae*. J Antimicrob Chemother 72:3035–3042. doi:10.1093/jac/dkx234. 28961916 PMC5890713

[16] Liu Y-Y, Chandler CE, Leung LM, McElheny CL, Mettus RT, Shanks RMQ, Liu J-H, Goodlett DR, Ernst RK, Doi Y. 2017. Structural modification of lipopolysaccharide conferred by mcr-1 in Gram-negative ESKAPE pathogens. Antimicrob Agents Chemother 61. doi:10.1128/AAC.00580-17. PMC5444183 28373195

[17] Furniss RCD, Dortet L, Bolland W, Drews O, Sparbier K, Bonnin RA, Filloux A, Kostrzewa M, Mavridou DAI, Larrouy-Maumus G. 2019. Detection of colistin resistance in *Escherichia coli* by use of the MALDI Biotyper Sirius mass spectrometry system. J Clin Microbiol 57. doi:10.1128/JCM.01427-19. PMC6879293 31597744

[18] Pedersen MG, Olesen HV, Jensen-Fangel S, Norskov-Lauritsen N, Wang M. 2018. Colistin resistance in *Pseudomonas aeruginosa* and *Achromobacter spp*. cultured from Danish cystic fibrosis patients is not related to plasmid-mediated expression of mcr-1. J Cyst Fibros 17:e22–e23. doi:10.1016/j.jcf.2017.12.001. 29254822

[19] Clinical and Laboratory Standards Institute. 2018. Methods for dilution antimicrobial susceptibility tests for bacteria that grow aerobically, 11th ed. Clinical and Laboratory Standards Institute, Wayne, PA.

[20] Hindler JA, Humphries RM. 2013. Colistin MIC variability by method for contemporary clinical isolates of multidrug-resistant Gram-negative bacilli. J Clin Microbiol 51:1678–1684. doi:10.1128/JCM.03385-12. 23486719 PMC3716094

[21] Nation RL, Li J. 2009. Colistin in the 21st century. Curr Opin Infect Dis 22:535–543. doi:10.1097/QCO.0b013e328332e672. 19797945 PMC2869076

[22] Nation RL, Li J, Cars O, Couet W, Dudley MN, Kaye KS, Mouton JW, Paterson DL, Tam VH, Theuretzbacher U, Tsuji BT, Turnidge JD. 2015. Framework for optimisation of the clinical use of colistin and polymyxin B: the Prato polymyxin consensus. Lancet Infect Dis 15:225–234. doi:10.1016/S1473-3099(14)70850-3. 25459221

[23] Poirel L, Jayol A, Nordmann P. 2017. Polymyxins: antibacterial activity, susceptibility testing, and resistance mechanisms encoded by plasmids or chromosomes. Clin Microbiol Rev 30:557–596. doi:10.1128/CMR.00064-16. 28275006 PMC5355641

[24] Chew KL, La MV, Lin RTP, Teo JWP. 2017. Colistin and polymyxin B susceptibility testing for carbapenem-resistant and mcr-positive Enterobacteriaceae: comparison of Sensititre, MicroScan, Vitek 2, and Etest with broth microdilution. J Clin Microbiol 55:2609–2616. doi:10.1128/JCM.00268-17. 28592552 PMC5648698

[25] Behera B, Mathur P, Das A, Kapil A, Gupta B, Bhoi S, Farooque K, Sharma V, Misra MC. 2010. Evaluation of susceptibility testing methods for polymyxin. Int J Infect Dis 14:e596-601. doi:10.1016/j.ijid.2009.09.001. 20045367

[26] European Centre for Disease Prevention and Control. 2019. Laboratory manual for carbapenem and colistin resistance detection and characterisation for the survey of carbapenem- and/or colistin-resistant Enterobacteriaceae – Version 2.0. ECDC, Stockholm.

[27] The European Committee on Antimicrobial Susceptibility Testing. 2021. Breakpoint tables for interpretation of MICs and zone diameters. Version 11.0. https://www.eucast.org/ast_of_bacteria/previous_versions_of_documents/.

[28] Hou TY, Chiang-Ni C, Teng SH. 2019. Current status of MALDI-TOF mass spectrometry in clinical microbiology. J Food Drug Anal 27:404–414. doi:10.1016/j.jfda.2019.01.001. 30987712 PMC9296205

[29] Fournier PE, Drancourt M, Colson P, Rolain JM, Scola BL, Raoult D. 2013. Modern clinical microbiology: new challenges and solutions. Nat Rev Microbiol 11:574–585. doi:10.1038/nrmicro3068. 24020074 PMC7097238

[30] Bauer KA, Perez KK, Forrest GN, Goff DA. 2014. Review of rapid diagnostic tests used by antimicrobial stewardship programs. Clin Infect Dis 59 Suppl 3:S134–S145. doi:10.1093/cid/ciu547. 25261540

[31] Sorensen M, Chandler CE, Gardner FM, Ramadan S, Khot PD, Leung LM, Farrance CE, Goodlett DR, Ernst RK, Nilsson E. 2020. Rapid microbial identification and colistin resistance detection via MALDI-TOF MS using a novel on-target extraction of membrane lipids. Sci Rep 10:21536. doi:10.1038/s41598-020-78401-3. 33299017 PMC7725828

[32] Leung LM, Fondrie WE, Doi Y, Johnson JK, Strickland DK, Ernst RK, Goodlett DR. 2017. Identification of the ESKAPE pathogens by mass spectrometric analysis of microbial membrane glycolipids. Sci Rep 7:6403. doi:10.1038/s41598-017-04793-4. 28743946 PMC5526941

[33] Gharaibeh MH, Shatnawi SQ. 2019. An overview of colistin resistance, mobilized colistin resistance genes dissemination, global responses, and the alternatives to colistin: a review. Vet World 12:1735–1746. doi:10.14202/vetworld.2019.1735-1746. 32009752 PMC6925059

[34] Liu Y-Y, Wang Y, Walsh TR, Yi L-X, Zhang R, Spencer J, Doi Y, Tian G, Dong B, Huang X, Yu L-F, Gu D, Ren H, Chen X, Lv L, He D, Zhou H, Liang Z, Liu J-H, Shen J. 2016. Emergence of plasmid-mediated colistin resistance mechanism MCR-1 in animals and human beings in China: a microbiological and molecular biological study. Lancet Infect Dis 16:161–168. doi:10.1016/S1473-3099(15)00424-7. 26603172

[35] El-Sayed Ahmed MAE-G, Zhong L-L, Shen C, Yang Y, Doi Y, Tian G-B. 2020. Colistin and its role in the era of antibiotic resistance: an extended review (2000–2019). Emerg Microbes Infect 9:868–885. doi:10.1080/22221751.2020.1754133. 32284036 PMC7241451

[36] Mushtaq S, Reynolds R, Gilmore MC, Esho O, Adkin R, Garcia-Romero I, Chaudhry A, Horner C, Bartholomew TL, Valvano MA, Dry M, Murray J, Pichon B, Livermore DM. 2020. Inherent colistin resistance in genogroups of the *Enterobacter cloacae* complex: epidemiological, genetic and biochemical analysis from the BSAC Resistance Surveillance Programme. J Antimicrob Chemother 75:2452–2461. doi:10.1093/jac/dkaa201. 32514538

[37] Davin-Regli A, Pages JM. 2015. *Enterobacter aerogenes* and *Enterobacter cloacae*; versatile bacterial pathogens confronting antibiotic treatment. Front Microbiol 6:392. doi:10.3389/fmicb.2015.00392. 26042091 PMC4435039

[38] Chavda KD, Chen L, Fouts DE, Sutton G, Brinkac L, Jenkins SG, Bonomo RA, Adams MD, Kreiswirth BN. 2016. Comprehensive genome analysis of carbapenemase-producing Enterobacter spp.: new insights into Phylogeny, population structure, and resistance mechanisms. mBio 7. doi:10.1128/mBio.02093-16. PMC5156309 27965456

[39] Moskowitz SM, Ernst RK, Miller SI. 2004. PmrAB, a two-component regulatory system of Pseudomonas aeruginosa that modulates resistance to cationic antimicrobial peptides and addition of aminoarabinose to lipid A. J Bacteriol 186:575–579. doi:10.1128/JB.186.2.575-579.2004. 14702327 PMC305751

[40] Yusuf E, van Westreenen M, Goessens W, Croughs P. 2020. The accuracy of four commercial broth microdilution tests in the determination of the minimum inhibitory concentration of colistin. Ann Clin Microbiol Antimicrob 19:42. doi:10.1186/s12941-020-00383-x. 32928253 PMC7488655

[41] Satlin MJ, Lewis JS, Weinstein MP, Patel J, Humphries RM, Kahlmeter G, Giske CG, Turnidge J. 2020. Clinical and Laboratory Standards Institute and European Committee on Antimicrobial Susceptibility Testing Position Statements on polymyxin B and colistin clinical breakpoints. Clin Infect Dis 71:e523–e529. 32052041 10.1093/cid/ciaa121

[42] Ezadi F, Ardebili A, Mirnejad R. 2019. Antimicrobial susceptibility testing for polymyxins: challenges, issues, and recommendations. J Clin Microbiol 57. doi:10.1128/JCM.01390-18. PMC6440778 30541939

[43] Simar S, Sibley D, Ashcraft D, Pankey G. 2017. Colistin and polymyxin B minimal inhibitory concentrations determined by Etest found unreliable for Gram-negative bacilli. Ochsner J 17:239–242. 29026355 PMC5625981

[44] Landman D, Salamera J, Quale J. 2013. Irreproducible and uninterpretable polymyxin B MICs for Enterobacter cloacae and Enterobacter aerogenes. J Clin Microbiol 51:4106–4111. doi:10.1128/JCM.02129-13. 24088860 PMC3838098

[45] Falagas ME, Makris GC, Dimopoulos G, Matthaiou DK. 2008. Heteroresistance: a concern of increasing clinical significance? Clin Microbiol Infect 14:101–104. doi:10.1111/j.1469-0691.2007.01912.x. 18093235

[46] Band VI, Crispell EK, Napier BA, Herrera CM, Tharp GK, Vavikolanu K, Pohl J, Read TD, Bosinger SE, Trent MS, Burd EM, Weiss DS. 2016. Antibiotic failure mediated by a resistant subpopulation in Enterobacter cloacae. Nat Microbiol 1:16053. doi:10.1038/nmicrobiol.2016.53. 27572838 PMC5154748

[47] Band VI, Weiss DS. 2019. Heteroresistance: a cause of unexplained antibiotic treatment failure? PLoS Pathog 15:e1007726. doi:10.1371/journal.ppat.1007726. 31170271 PMC6553791

[48] Band VI, Satola SW, Smith RD, Hufnagel DA, Bower C, Conley AB, Rishishwar L, Dale SE, Hardy DJ, Vargas RL, Dumyati G, Kainer MA, Phipps EC, Pierce R, Wilson LE, Sorensen M, Nilsson E, Jordan IK, Burd EM, Farley MM, Jacob JT, Ernst RK, Weiss DS. 2021. Colistin heteroresistance is largely undetected among carbapenem-resistant Enterobacterales in the United States. mBio 12. doi:10.1128/mBio.02881-20. PMC7858057 33500343

[49] Tam VH, Schilling AN, Vo G, Kabbara S, Kwa AL, Wiederhold NP, Lewis RE. 2005. Pharmacodynamics of polymyxin B against Pseudomonas aeruginosa. Antimicrob Agents Chemother 49:3624–3630. doi:10.1128/AAC.49.9.3624-3630.2005. 16127031 PMC1195418

[50] Ordooei Javan A, Shokouhi S, Sahraei Z. 2015. A review on colistin nephrotoxicity. Eur J Clin Pharmacol 71:801–810. doi:10.1007/s00228-015-1865-4. 26008213

[51] Fernandez L, Gooderham WJ, Bains M, McPhee JB, Wiegand I, Hancock RE. 2010. Adaptive resistance to the “last hope” antibiotics polymyxin B and colistin in Pseudomonas aeruginosa is mediated by the novel two-component regulatory system ParR-ParS. Antimicrob Agents Chemother 54:3372–3382. doi:10.1128/AAC.00242-10. 20547815 PMC2916309

[52] Liang T, Schneider T, Yoon SH, Oyler BL, Leung LM, Fondrie WE, Yen G, Huang Y, Ernst RK, Nilsson E, Goodlett DR. 2018. Optimized surface acoustic wave nebulization facilitates bacterial phenotyping. Int J Mass Spectrometry 427:65–72. doi:10.1016/j.ijms.2017.09.007.

