# Supplementary material for: A Novel Lipid-Based MALDI-TOF Assay for the Rapid Detection of Colistin-Resistant Enterobacter Species
Source: Microbiol Spectr. 2022 Feb 2;10(1):e01445-21. doi: 10.1128/spectrum.01445-21 (PMC8809348; doi:10.1128/spectrum.01445-21)
Supplement: SUPPLEMENTAL FILE 1 — Supplemental material. Download SPECTRUM01445-21_Supp_1_seq8.pdf, PDF file, 0.2 MB [file spectrum01445-21_supp_1_seq8.pdf]

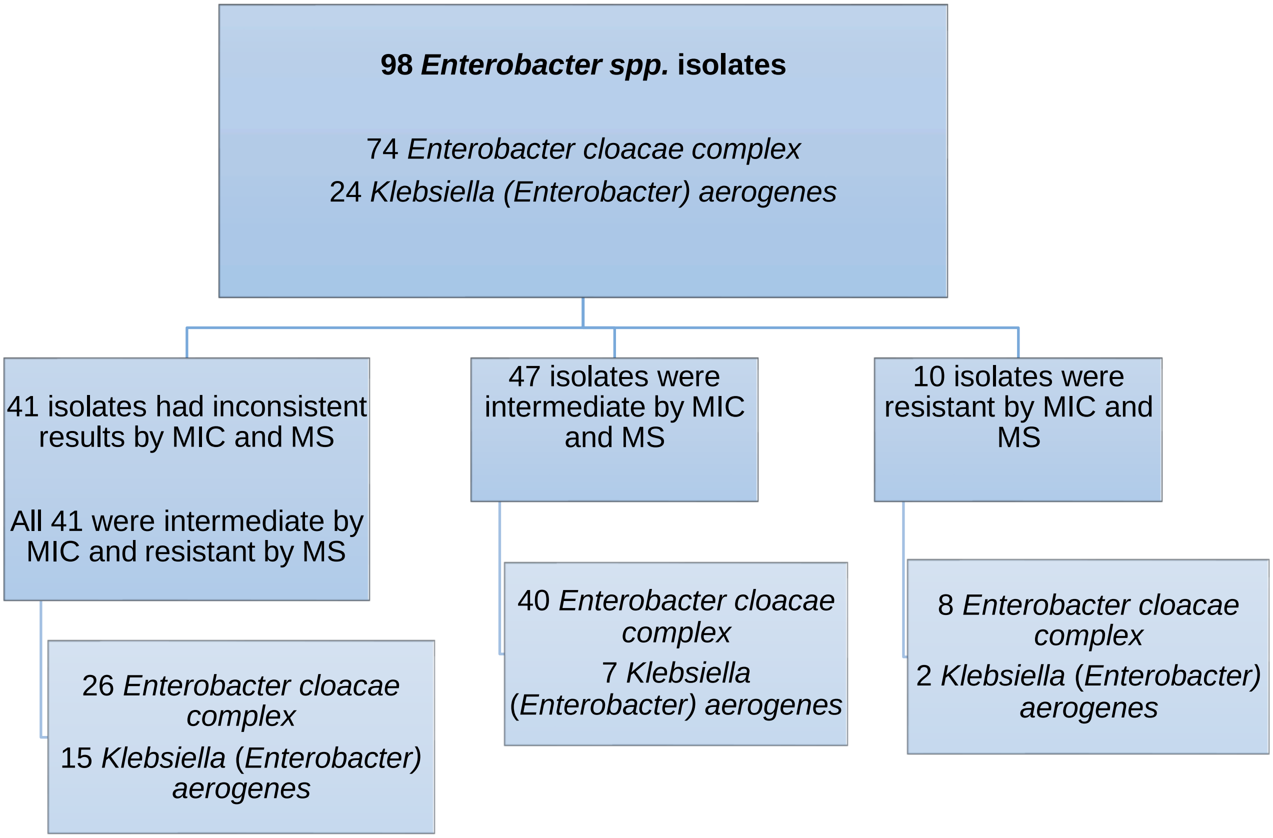

Figure S1. Schematic flow chart demonstrating breakdown of species, MIC, and MS results. MIC and MS results were further broken down by species.

Isolate	MIC	Ara4N	Kill 50 (ug/mL)	Biotyper ID	Infection Site
1069	0.5	x	1.65	*Klebsiella aerogenes*	Sputum
1096	1	x	1.76	*Enterobacter cloacae complex*	Urine
1137	1		0	*Enterobacter cloacae complex*	Urine
1192	0.5	x	0.73	*Klebsiella aerogenes*	Sputum
1194	0.5	x	2.29	*Klebsiella aerogenes*	Blood
1354	1	x	5.5	*Enterobacter cloacae complex*	Blood
1489	0.25	x	0.92	*Klebsiella aerogenes*	Tissue
1556	0.5	x	0.98	*Enterobacter cloacae complex*	Bronchoalveolar lavage
1749	0.5	x	0.93	*Klebsiella aerogenes*	Bronchoalveolar lavage
1813	0.5		0.36	*Enterobacter cloacae complex*	Wound
1869	1	x	0.92	*Enterobacter cloacae complex*	Trach aspirate
1904	0.5		0	*Enterobacter cloacae complex*	Drainage left upper quadrant
1914	0.5	x	0.59	*Enterobacter cloacae complex*	Neck tissue
1947	0.5	x	0.97	*Enterobacter cloacae complex*	Urine
1955	0.5	x	0.61	*Enterobacter cloacae complex*	Urine
1958	0.5		0.44	*Enterobacter cloacae complex*	Urine
1968	2		0.09	*Enterobacter cloacae complex*	Urine
YDC560-1	0.125		0	*Enterobacter cloacae complex*	Nasal pharynx
YDC578	0.5		0	*Enterobacter cloacae complex*	Trach aspirate
YDC772-1	1		0.05	*Klebsiella aerogenes*	Urine
YDC455	1		0	*Enterobacter cloacae complex*	Pigtail drainage
YDC456	2		0	*Enterobacter cloacae complex*	Jackson-Pratt surgical drainage
YDC469-1	4	x	3.86	*Enterobacter cloacae complex*	Drainage left upper quadrant
YDC469-2	2		1.09	*Enterobacter cloacae complex*	Drainage left upper quadrant
YDC477	1		0	*Enterobacter cloacae complex*	Blood
YDC482	2		0	*Enterobacter cloacae complex*	Blood
YDC492	0.25		0	*Enterobacter cloacae complex*	Bronchoalveolar lavage
YDC497	0.5		1.11	*Klebsiella aerogenes*	Sputum
YDC500	0.5	x	2.42	*Klebsiella aerogenes*	Bronchoalveolar lavage
YDC503	1		0	*Enterobacter cloacae complex*	Abdominal incision
YDC504	0.5	x	3.04	*Enterobacter cloacae complex*	Groin hematoma
YDC506	2		0	*Enterobacter cloacae complex*	Neck tissue

YDC516	64	x	29.3	*Enterobacter cloacae complex*	Urine
YDC518	1		0	*Enterobacter cloacae complex*	Blood
YDC520	0.125		0.23	*Enterobacter cloacae complex*	Urine
YDC531	0.125		0	*Klebsiella aerogenes*	Urine
YDC560 -2	0.25		0.21	*Enterobacter cloacae complex*	Cerebrospinal fluid
YDC561	2	x	0.76	*Enterobacter cloacae complex*	Blood
YDC567	0.5		0	*Enterobacter cloacae complex*	Bronchoalveolar lavage
YDC573	0.5	x	5.77	*Enterobacter cloacae complex*	Urine
YDC588	0.125	x	5.15	*Klebsiella aerogenes*	Trach aspirate
YDC590	0.5	x	1.32	*Enterobacter cloacae complex complex*	Bronchial wash
YDC592	0.25		0	*Enterobacter cloacae complex*	Hepatic fluid
YDC596	0.5		0.63	*Enterobacter cloacae complex*	Blood
YDC597	0.5		0	*Enterobacter cloacae complex*	Deep wound
YDC598	128	x	1	*Klebsiella aerogenes*	Urine
YDC599	2	x	4.75	*Enterobacter cloacae complex complex*	Hip acetabulum
YDC600	1		0.13	*Enterobacter cloacae complex*	Bile
YDC601	1	x	0.52	*Enterobacter cloacae complex*	Bronchoalveolar lavage
YDC602	0.125		0	*Enterobacter cloacae complex*	abdominal fistula
YDC607	0.125		0.44	*Enterobacter cloacae complex*	Urine
YDC611	0.5	x	1.67	*Enterobacter cloacae complex*	Urine
YDC612	0.5		0.49	*Enterobacter cloacae complex*	Bronchoalveolar lavage
YDC620	1		0	*Enterobacter cloacae complex*	Urine
YDC621	1		0	*Enterobacter cloacae complex*	Bronchial wash
YDC624	0.5	x	4.41	*Enterobacter cloacae complex*	Urine
YDC634	0.125		0	*Klebsiella aerogenes*	Urine
YDC635 -1	0.25		1.26	*Enterobacter cloacae complex*	Wound
YDC640	0.5	x	0.62	*Klebsiella aerogenes*	Urine
YDC649	0.125		0	*Enterobacter cloacae complex*	Blood
YDC650	0.5		0	*Enterobacter cloacae complex*	Blood
YDC655	0.5	x	0.53	*Klebsiella aerogenes*	Upper abdomen drainage
YDC660	128	x	5.58	*Enterobacter cloacae complex*	Tissue
YDC665	0.25		0	*Enterobacter cloacae complex*	Bronchial wash
YDC666	128	x	9.49	*Klebsiella aerogenes*	abdominal hematoma
YDC668	0.125		0	*Klebsiella aerogenes*	Urine
YDC671	1	x	2.43	*Enterobacter cloacae complex*	Hernia repair wound

YDC673	0.25		0.42	*Enterobacter cloacae complex*	Perigastric fluid
YDC675	32	x	1.06	*Enterobacter cloacae complex*	Urine
YDC678-1	0.5		0	*Enterobacter cloacae complex*	Abdominal abscess
YDC678-2	0.5		0	*Enterobacter cloacae complex*	Tissue
YDC678-3	0.5		0	*Enterobacter cloacae complex*	Deep wound
YDC704	64	x	1.79	*Enterobacter cloacae complex*	Bronchoalveolar lavage
YDC710	128	x	0.63	*Enterobacter cloacae complex*	Wound
YDC711	1	x	1.24	*Enterobacter cloacae complex*	Body fluid
YDC712	2		0.14	*Enterobacter cloacae complex*	Abdominal tissue
YDC732	0.25	x	0.51	*Klebsiella aerogenes*	Bronchoalveolar lavage
YDC740	1	x	8.65	*Enterobacter cloacae complex*	Bronchoalveolar lavage
YDC743	1	x	9.66	*Klebsiella aerogenes*	Bronchoalveolar lavage
YDC744	1	x	2.69	*Klebsiella aerogenes*	Sputum
YDC746	1		0	*Enterobacter cloacae complex*	Blood
YDC750	1	x	2.12	*Klebsiella aerogenes*	Pancreas tissue
YDC751	0.25	x	1.94	*Klebsiella aerogenes*	Right heel tissue
YDC753	0.25		0	*Enterobacter cloacae complex*	Blood
YDC763-2	1		0.43	*Enterobacter cloacae complex*	Bronchoalveolar lavage
YDC766	1	x	5.6	*Enterobacter cloacae complex*	Sputum
YDC769	0.25	x	5.25	*Enterobacter cloacae complex*	Jackson-Pratt surgical drain
YDC783	0.5	x	13.46	*Enterobacter cloacae complex*	Upper respiratory tract
YDC791	0.25		0.22	*Klebsiella aerogenes*	Bronchial wash
YDC792	0.125		1	*Klebsiella aerogenes*	Upper respiratory tract
YDC794	128	x	6.49	*Enterobacter cloacae complex*	Bronchoalveolar lavage
YDC796	0.25	x	4	*Enterobacter cloacae complex*	Blood
YDC470	1	x	Higher then tested	*Enterobacter cloacae complex*	Drainage left upper quadrant
YDC575	128	x	Higher then tested	*Enterobacter cloacae complex*	Bronchoalveolar lavage
YDC603	0.5	x	26	*Enterobacter cloacae complex*	Urine
YDC772-2	2	x	Higher then tested	*Klebsiella aerogenes*	Urine
YDC773	0.5	x	Higher then tested	*Enterobacter cloacae complex*	Bronchoalveolar lavage

YDC589	0.25	x	Higher then tested	*Enterobacter cloacae complex*	Sputum

Table S1. Comprehensive table showing all 98 isolates in this study including MIC, whether they have Ara4N, $Kill_{50}$, Biotyper identification, and infection site where bacteria was isolated.
